# An adaptive method for determining the optimal number of topics in topic modeling

**DOI:** 10.7717/peerj-cs.2723

**Published:** 2025-02-28

**Authors:** Yang Xu, Yueyi Zhang, Yefang Sun, Hanting Zhou

**Affiliations:** 1 College of Economics and Management, China Jiliang University, Hangzhou, Zhejiang, China; 2 College of Economics and Management, China Jiliang University College of Modern Science and Technology, Yiwu, Zhejiang, China; 3 School of Management, Hangzhou Dianzi University, Hangzhou, Zhejiang, China

**Keywords:** Topic modeling, Inter-class distance, AICDR, Optimal number of topics

## Abstract

Topic models have been successfully applied to information classification and retrieval. The difficulty in successfully applying these technologies is to select the appropriate number of topics for a given *corpus*. Selecting too few topics can result in information loss and topic omission, known as underfitting. Conversely, an excess of topics can introduce noise and complexity, resulting in overfitting. Therefore, this article considers the inter-class distance and proposes a new method to determine the number of topics based on clustering results, named average inter-class distance change rate (AICDR). AICDR employs the Ward’s method to calculate inter-class distances, then calculates the average inter-class distance for different numbers of topics, and determines the optimal number of topics based on the average distance change rate. Experiments show that the number of topics determined by AICDR is more in line with the true classification of datasets, with high inter-class distance and low inter-class similarity, avoiding the phenomenon of topic overlap. AICDR is a technique predicated on clustering results to select the optimal number of topics and has strong adaptability to various topic models.

## Introduction

Modern information systems generate a huge number of texts such as policies, news, and comments. Analysis of big data is impossible without the construction of formalized mathematical models (Ignatenko et al., 2018). Topic models are gaining popularity in social sciences (Peng & Zhang, 2024). Models such as non-negative matrix factorization (Kekere, Marivate & Hattingh, 2023) and Latent Dirichlet Allocation (LDA) (Ding, Kang & Ren, 2024). These techniques rely on statistical modeling to mine the semantic information implied in large-scale text datasets and classify massive texts according to different topics, and each text cluster is interpreted by different topic words (Zhao et al., 2018).

In topic modeling, the determination of the number of topics (parameter $K$) is crucial for text analysis. Identification of the optimum number of topics is one of the main challenges for the existing methods for topic modeling. Choosing too few topics will result in overly broad topics, loss of information and omission of topics, while choosing too many will result in the over-clustering of a *corpus* into many small, noisy and highly-similar topics (Greene, O’Callaghan & Cunningham, 2014), and the topics are prone to overlap and merge. Thus, the selection of $K$ is particularly important.

Perplexity is a commonly used measurement in information theory to characterize topic quality, with lower perplexity denoting a better probabilistic model (Huang, Ma & Chen, 2017). To solve the problem of multiple elbows in perplexity, a new method for calculating the rate of change of perplexity (Zhao et al., 2015) is used to determine the appropriate number of topics. Similarly, coherence is an indicator used to evaluate the quality of a topic and is also applicable to the selection of the number of topics (O’Callaghan et al., 2015). It evaluates the frequency of word co-occurrence and measures the correlation between words in a topic. Perplexity and coherence are mostly used in probabilistic topic models, which have limited adaptability.

The classical probabilistic topic models (LDA) are widely used in topic classification tasks (Chen et al., 2017). Therefore, most studies use probabilistic topic models to research the selection of topic numbers. A density-based adaptive LDA model selection approach that integrates the concept of density clustering to adaptively determine an appropriate number of topics (Cao et al., 2009; Lu et al., 2013; Wang et al., 2019). Some improvements or combinations of metrics or evaluation indicators are equally applicable to this task. An improved online LDA (IOLDA) uses Jensen–Shannon (JS) scatter to calculate the association between topics and screen out similar topics, and the JS scatter is smaller when the number of topics is close to the optimal value (He, Chen & Du, 2015). The combination of JS divergence and perplexity is used to select the optimal number of topics, which improves the problem of using the perplexity formula alone (Peng & Yuefen, 2016). A comprehensive index of perplexity, isolation, stability, and consistency is constructed, which can effectively determine the optimal number of topics in the LDA model (Gan & Qi, 2021). The combination of stability and coherence is also applicable to neural topic models to select the number of topics (Koltcov et al., 2024).

To reduce the reliance on probability distributions or topic-term matrices, a stability analysis significantly enhances its versatility, rendering it applicable to topic models and classification methods (Greene, O’Callaghan & Cunningham, 2014). The elbow method is a commonly used technique for determining the optimal number of clusters in K-Means clustering. Its core idea is to identify the best number of clusters by observing the relationship between the sum of squared errors (SSE) of the clustering results and different K (Liu & Deng, 2021; Shi et al., 2021). The silhouette method is another well-known method with decent performance in estimating the potential optimal cluster number (Arbelaitz et al., 2013; Rodriguez et al., 2019). Similarly, it’s not uncommon to use metrics to evaluate the optimal cluster number (Ding, Tarokh & Yang, 2017; Zheng et al., 2023), they may also be applied to topic models.

The optimal number of topics should produce a good clustering result, *i.e*., high similarity of texts within topics and low similarity of texts within topics. In the literature, most methods for selecting the number of topics rely on probability distributions. For non-probabilistic topic models, these methods are difficult to apply. Motivated by this, this article proposes a new method for determining the number of topics in topic models based on inter-class distance, named average inter-class distance change rate (AICDR). AICDR calculates the diameters for each class and the merged class, then derives the inter-class distance as the square root of the diameter difference between them. It subsequently computes the average inter-class distance across varying numbers of topics and identifies the optimal number of topics based on the average inter-class distance change rate. AICDR is not bound by the constraints of the topic models, and the selection of the optimal number of topics is done only through the clustering results. The inter-class distance between topics is calculated based on Ward’s method (Murtagh & Legendre, 2014), the number of topics corresponding to the maximum AICDR is the optimal number of topics. To verify the feasibility and adaptability of the proposed method, this research compares stability analysis and elbow method to select the optimal K value. The contributions in this article are summarized as follows:

- Ward’s method is a method of hierarchical clustering that aims to produce classes by minimizing the intra-class variance. It merges the two classes with the smallest sum of distances (sum of squared deviations) until the condition is satisfied. We define the inter-class distance as the square root of the difference between the diameter of the merged class and the diameter of the original classes. The sum of the Euclidean distances from all objects in a class to the class mean is defined as the diameter of the class (*i.e*., the squared deviation). The diameter of class indicates the compactness of the sample, while the distance between classes indicates the degree of separation between classes.
- A new method for determining the number of topics is proposed, named AICDR. AICDR considers inter-class similarity and intra-class similarity comprehensively, the $K$ corresponding to its maximum is the optimal number of topics, which avoids topic overlap. Mainly, it is not limited by topic models or clustering methods and has good adaptability to most methods.
- Through experiments on several public datasets, the feasibility of the proposed method is fully verified, which provides a useful reference for similar research.

The remainder of this article is organized as follows. “Related Work” introduces the previous research on topic models; “Methods” describes the principles of several topic models used in experiments, the formulaic definition of AICDR, and inter-class distance; “The Workflow of AICDR” introduces the process of selecting the optimum number of topics; “Experiments” talks about the experimental results and analysis; “Conclusion” concludes the article and proposes the future work.

## Related work

Generally, topic modeling methods are mainly classified as probabilistic and non-probabilistic in the literature (Kherwa & Bansal, 2018). This section reviews three more detailed branches used for developing topic modeling algorithms: probabilistic topic models, matrix factorization-based topic models and neural topic models.

### Probabilistic topic models

Introduced as an initial probabilistic approach to topic modeling, probabilistic latent semantic analysis (PLSA) has its limitations as it fixes the distributions of topics and words within a document. LDA (Blei, Ng & Jordan, 2003) addresses this by applying Dirichlet priors to the distributions, thus allowing for a probabilistic assignment of topics and words. LDA has shown remarkable efficacy, attracting persistent research attention (Altarturi, Saadoon & Anuar, 2023).

Due to the limited co-occurrence information of words in short texts, traditional long-text topic modeling algorithms (*e.g*., PLSA and LDA) based on word co-occurrences cannot solve this problem very well (Qiang et al., 2020). A collapsed Gibbs Sampling algorithm for the Dirichlet multinomial mixture (GSDMM) (Yin & Wang, 2014) model for short text clustering defaults to all words in the document following a topic. WV+GSDMMK (Agarwal, Sikka & Awasthi, 2024) improves service-to-topic mapping by determining semantic similarity among features, and K-means clustering is applied on service to topic representation. Biterm topic model (BTM) (Cheng et al., 2014) addresses the challenge of short text topic modeling by directly modeling the generation of word co-occurrence patterns, or biterms, throughout the *corpus*. Therefore, a word co-occurrence network-based model (WNTM) (Zuo, Zhao & Xu, 2016) represents the word co-occurrence network back to a pseudo-document set, where a word forms a pseudo document with adjacent words and models the thematic distribution of each word. Similarly, pseudo-document-based topic model (PTM) (Zuo et al., 2021), also utilizes word co-occurrence information to construct pseudo-document and topic modeling.

### Matrix factorization-based topic models

Latent semantic indexing (LSI) (Papadimitriou et al., 1998) uses singular value decomposition techniques to capture the latent semantic relationships between words, while non-negative matrix factorization (NMF) approximates the reconstruction of the original matrix by decomposing the data matrix into two non-negative matrices (Xu, Liu & Gong, 2003). Compared to LSI, the advantage of NMF lies in its non-negativity and sparsity constraints, making the decomposition results easier to interpret. NMF has been successfully applied to topic modeling and text clustering, due to its superior performance in clustering high-dimensional data (Carbonetto et al., 2022).

To continuously improve the performance of NMF, some scholars have considered constructing graphs, *i.e*. data graph and feature graph, to explore the geometric structure of data manifold and feature manifold (Cai et al., 2011; Gu & Zhou, 2009). Semantic information can also be embedded in NMF to adapt to short texts. Semantics-assisted NMF (SeaNMF) (Shi et al., 2018) effectively incorporates word-context semantic correlations into the model. Word co-occurrence regularized NMF (WC-NMTF) (Salah et al., 2018) maps frequently co-occurring words roughly to the same direction in the latent space to reflect the relationships between them. Both SeaNMF and WC-NMTF use point-wise mutual information (PMI) (Levy & Goldberg, 2014) Neighbourhood assistance-based NMF (NaNMF) (Athukorala & Mohotti, 2022) introduces text similarity as a regularization constraint to improve classification performance. Regularized asymmetric NMF (RANMF) (Aghdam & Zanjani, 2021) is the same way. The incorporation of regularization constraints (*i.e*., regularizers) in NMF is an effective approach. NMF-WR (Li et al., 2024) integrates the Wasserstein metric into the NMF framework to enhance semantic representation and improve the reliability and interpretability of text embeddings.

### Neural topic models

Recent advances in neural variational inference have spawned a renaissance in deep latent variable models (Miao, Yu & Blunsom, 2015). Unlike traditional Bayesian topic models (*e.g*., PLSA and LDA), neural topic models use deep neural networks to approximate the intractable marginal distribution and thus gain strong generalization ability.

Using neural methods to replace providing parameterizable distributions on topics, permits training by backpropagation in the framework of neural variational (Miao, Grefenstette & Blunsom, 2017). Autoencoding variational inference for topic model (AVITM) (Srivastava & Sutton, 2017) introduces an autoencoder to approximate the posterior distribution, improving efficiency and accuracy. Neural variational gaussian mixture topic model (NVGMTM) (Tang et al., 2022) uses Gaussian distribution to depict the semantic relevance between words in the topics, each topic is considered as a multivariate Gaussian distribution over words in the word-embedding space. To fully utilize the discreteness of the topic space, the discrete-variational-inference-based topic model (DVITM) (Gupta & Zhang, 2023), learns dense topic embeddings homomorphic to word embeddings *via* discrete variational inference. Self-attention mechanism can capture the dependency relationships within the sequence (Vaswani et al., 2017). Therefore, topic attention model (TAM) (Wang & Yang, 2020) utilizes document-specific topic proportions and global topic vectors learned from neural topic model in the attention mechanism.

## Methods

### Topic models

#### LDA

LDA is a probabilistic statistical model for mining topic distributions and word distributions in document collections, identifying topic information hidden in document collections or *corpus*. The basic idea in LDA is that each document is represented as a probability distribution over hidden topics, while each topic is characterized as a probability distribution over some words. The generative process of the LDA model for each document is written as follows:

1) Draw each topic parameter $\beta_k \sim Dirichlet\; \left({\Phi} \right)$, for each topic $k\; \epsilon \; \left[ {1, \ldots K} \right].$
2) For each document $d\; \epsilon \; D$:
  a) Sample a topic distribution ${\theta }\sim{\rm \; }Dirichlet{\rm \; }\left( a \right)$
  b) For each of the *N* words ${w_n}$:

Sample a topic ${z_{m,n}}\; \sim Multionmial \;\left( {{{\theta}_m}} \right)$

Sample a word $w{\rm \; }\sim{\rm \; }Multionmial{\rm \; }\left( {{\beta _{\rm k}}{\rm \; }} \right)$ from $p({w_n}|{z_{m,n}},{\beta _k})$

**Figure 1:**
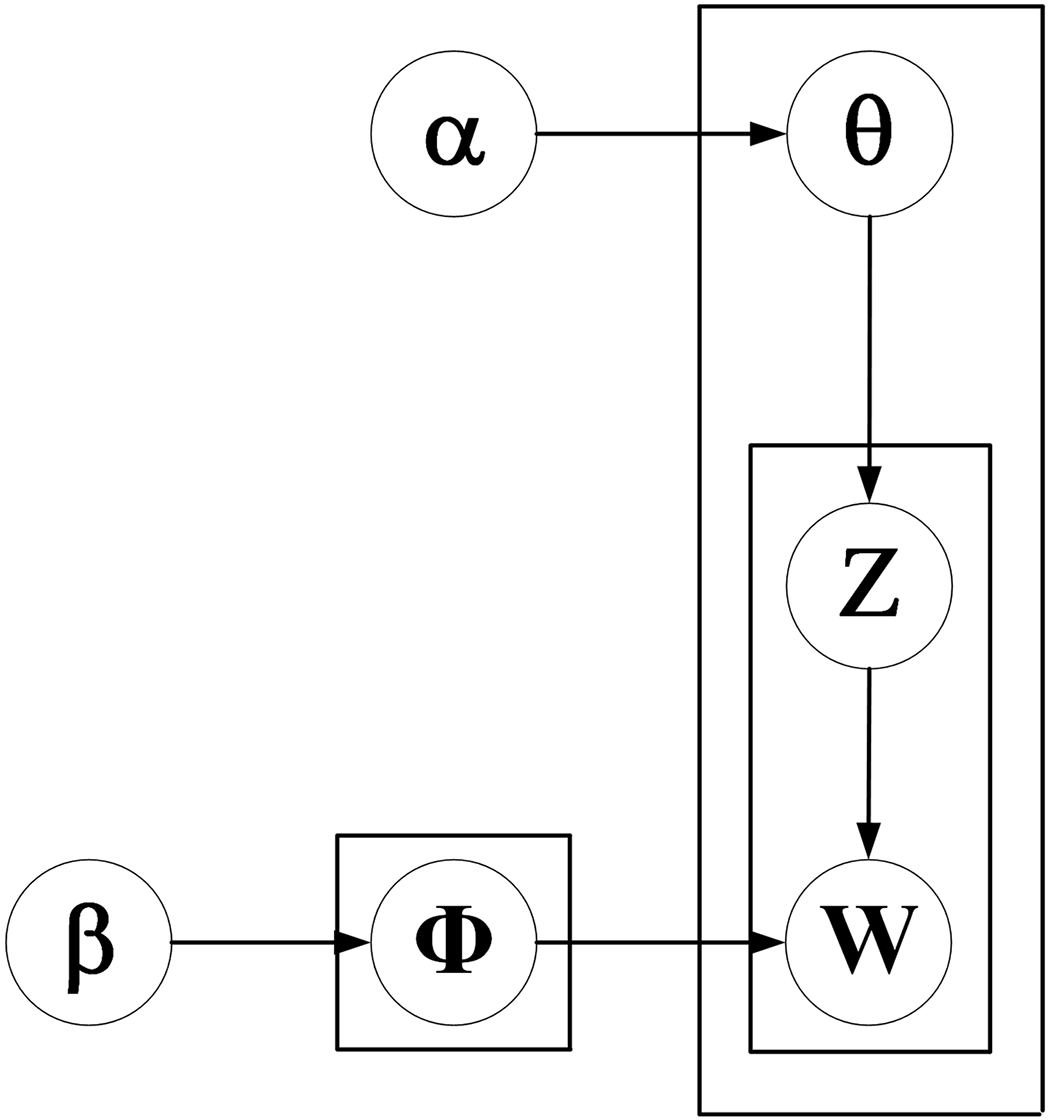
Graphical model representation of LDA.

In Fig. 1, *Φ* represents word distribution, $\theta$ represents topic distribution. α is a parameter of the prior distribution of topic distribution $\theta$ and *β* is a parameter of the prior distribution of word distribution. $Z$ and $W$ represent the distribution of document topic and word topic, respectively.

#### GSDMM

Dirichlet multinomial mixture (DMM) respectively chooses Dirichlet distribution for topic-word distribution ${\Phi}$ and document-topic distribution $\theta$ as prior distribution with parameter *α* and *β*. DMM samples a topic $Z$ for the document by multinomial distribution $\theta$, and then generates all words in the document from topic $Z$ by multinomial distribution ${\Phi}$. The generative process for DMM is described as follows:

1) Sample a topic distribution $\theta \sim Dirichlet{\rm \; }\left( a \right)$.
2) For each topic $k\; \epsilon \; \left[ {1, \ldots K} \right]\!:$
3) For each document $d\; \epsilon \; D$:
  a) Sample a topic ${\rm \; }{z_d}\sim Multionmial{\rm \; }\left( \theta \right)$
  b) For each word $w\; \epsilon \; \left[ {{w_{d,1}}, \ldots ,\; {w_{d,{n_d}}}} \right]$:

Sample a word $w \sim \; Multionmial{\rm \; }\left( {{{\Phi} _{{z_d}}}} \right).$

**Figure 2:**
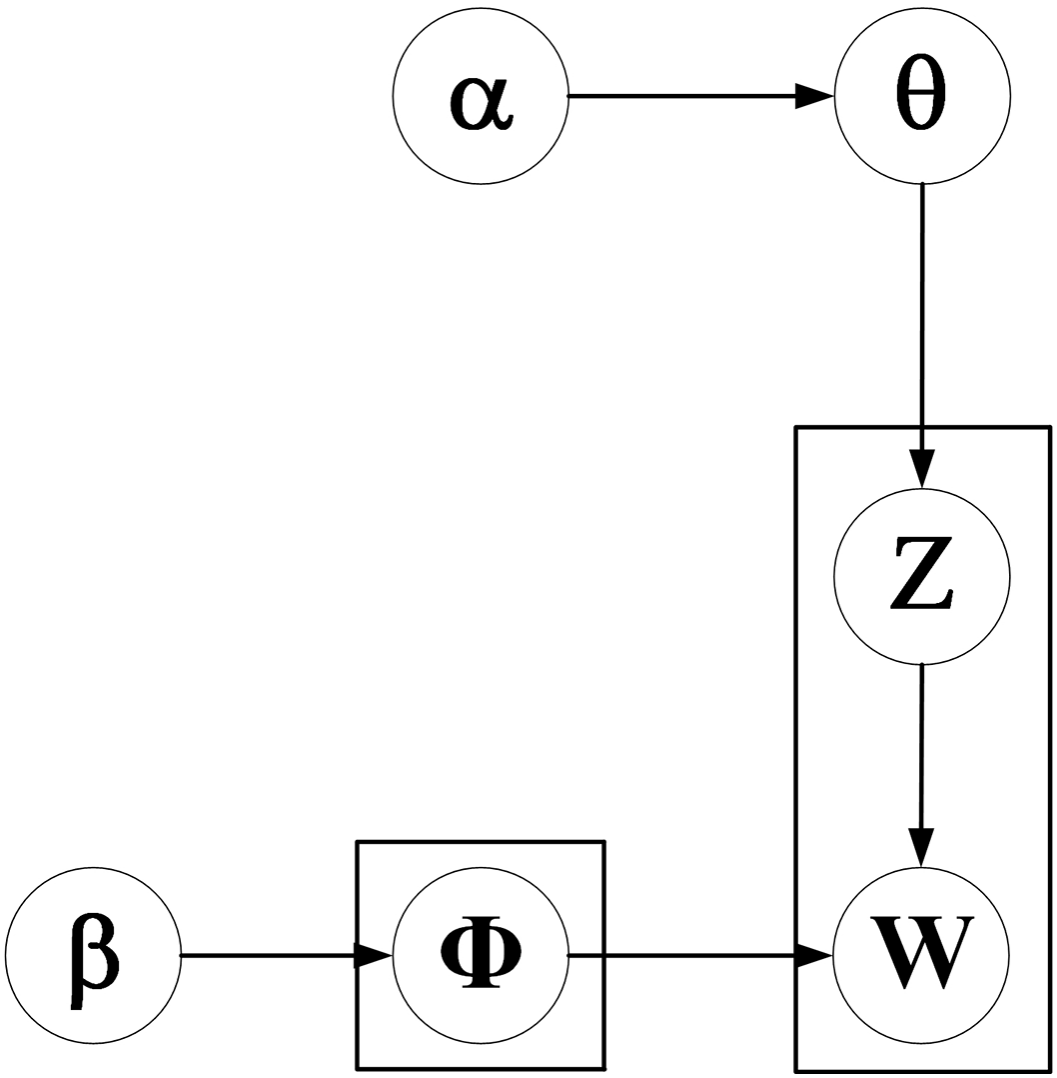
Graphical model representation of GSDMM.

The graphical model representation of GSDMM is shown in Fig. 2. Gibbs sampling algorithm for DMM (GSDMM) assumes that each text is sampled by a single topic.

#### NMF

The NMF method has been successfully applied to topic modeling, due to its superior performance in clustering high-dimensional data. A text dataset can be represented by a matrix $X{\rm \; }\epsilon {\rm \; }R_ + ^{{\rm \; \; }m \times n}$. $X$ approximates through two matrices $W\; \epsilon \; R_ + ^{\; \; m \times k}$ and $H{\rm \; }\epsilon {\rm \; }R_ + ^{{\rm \; \; }n \times k}$, *i.e*., $X \approx W{H^T}$. The NMF formula is as follows:

(1) $$\eqalign {F =\; & min\; \left| {\left| {X - W{H^T}} \right|} \right|\; _F^2 \cr & s.t.\; W \ge 0,\; H \ge 0.}$$

The index of the maximum value for each row $argmax\mathop \sum \nolimits_i^m {W_{\left( {i,:} \right)}}$ represents the topic of the *i-th* document. Similarly, $argmax\mathop \sum \nolimits_j^n {H_{\left( {j,:} \right)}}$ represents the topic of the *j-th* word.

#### SeaNMF

SeaNMF effectively incorporates the word-context semantic correlations into the model, where the semantic relationships between the words and their contexts are learned from the skip-gram view of the *corpus*. These correlations can be viewed as an alternative form of the word co-occurrence. It can overcome the problem that arises due to the data sparsity. Therefore, its objective function is as follows:

(2) $$\eqalign{F =\ & min{\rm \; }\left| {\left| {X - W{H^T}{\rm \; }} \right|} \right|{\rm \; }_F^2 + {\rm \; }\alpha ||P - H{Q^T}||_F^2 \\& s.t. W \ge 0,\; H \ge 0,\; Q \ge 0,}$$

where $P$ represents positive pointwise mutual information (PPMI) and $P\; \epsilon \; R_ + ^{\; \; n \times n}$, and $Q$ is a randomly initialized factor matrix and $Q\; \epsilon \; R_ + ^{\; \; n \times k}$. SeaNMF incorporates word co-occurrence information as a regularization constraint to compensate for the sparsity of short text.

Table 1 outlines the specific characteristics of LDA, GSDMM, NMF, and SeaNMF.

**Table 1:** Description of topic models.

Models	Type	Applicability
LDA	Probabilistic generative	Long text
GSDMM	Probabilistic generative	Short text
NMF	Non-probabilistic	Long text
SeaNMF	Non-probabilistic	Short text

### The proposed AICDR

For unstructured document sets, both the document content and the number of relevant topics are unknown, and the best number of topics is unknown. An insufficient number of topics may lead to underfitting of the model, a higher number of topics could result in a model that is too complex, making topic overlap. It is necessary to select the appropriate number of topics. The best clustering result, with a specified number of topics, should exhibit high intra-cluster similarity within the same topic and lower inter-cluster similarity between different topics.

In this section, we introduce the proposed method for determining the number of topics, AICDR. When the value of AICDR is maximized, the corresponding number of topics is optimal. Using AICDR to determine the number of topics results in higher inter-class distance or lower inter-class similarity. Inter-class distance is used to describe the distance between different classes after categorization, which quantifies the degree of difference or similarity between different classes.

There are various methods for defining inter-class distance, including the single linkage method, complete linkage method, group average method, centroid method, and sum of squares method. The central method and sum of squares method consider the global features of the class. Ward’s method (Murtagh & Legendre, 2014) is a hierarchical clustering approach based on the idea of sum of squared deviation, which defines the distance between two populations by merging the differences in intra-group variances before and after. This is precisely sum of squares method’s definition of inter-class distance. We utilize Ward’s method (*i.e*., sum of squares method) to define the diameter of the class and the inter-class distance. Therefore, the steps of AICDR are as follows:

(1) Calculate the diameter of the class.

(3) $${D_p} = \mathop \sum \limits_{i = 1}^m \sqrt {{{\left( {{t_i}\; - \; \bar t\; } \right)}^T}\left( {{t_i}- \bar t\; } \right)} ,$$

(4) $$\bar t\; = \; \displaystyle{1 \over m}\; \mathop \sum \limits_{i = 1}^m {t_i}.$$

${D_p}$ represents the diameter of the *p-th* class, ${t_i}$ represents the text vector, and $\bar t{\rm \; }$ represents the mean text vector. Define the sum of the Euclidean distances from all objects in a class to the class mean as the diameter of the class (*i.e*., the sum of squared deviations). The ideal clustering result should be a greater intra-class similarity, *i.e*., a smaller intra-class distance, or a smaller diameter of the class.

(2) Calculate the inter-class distance.

(5) $$D\left( {p,q} \right) = \sqrt {{D_{p,q}} - \; {D_p} - \; {D_{q}}} .$$

${D_{p,q}}$ represents the diameter of the merged class, $D\left( {p,q} \right)$ represents the inter-class distance between the *p-th* class and the *q-th* class. Merge two classes into a single class and compute its diameter; the inter-class distance is then the square root of the difference between the new diameter and the sum of the diameters of the original classes. After classification, the inter-class similarity should be small, *i.e*. the inter-class is large.

(3) Calculate average inter-class distance.

(6) $$ave\_dis\left( k \right) = \displaystyle{1 \over {\displaystyle{{k\left( {k - 1} \right)} \over 2}}}\mathop \sum \limits_{p = 1}^k \mathop \sum \limits_{q = p + 1}^k D(p,q).$$

The $ave\_dis\left( k \right)$ denotes the average inter-class distance for a topic number of K. As the number of topics increases, the value of the $ave\_dis\left( k \right)$ decreases, indicating that the inter-class distance between topics decreases gradually. Although the average inter-class distance method may generate meaningful results in some cases, it is not stable and its value decreases as the number of topics increases.

(4) Calculate AICDR.

(7) $$AICDR(k)={abs}(ave\_dis(k+1)-ave\_dis(k))$$

The larger the value of AICDR, the more the increase (decrease) in the number of topics affects the structure, and the corresponding parameter K is the optimal number of topics.

The complete AICDR algorithm is found in Algorithm 1.

**Algorithm 1:** Algorithm for the proposed AICDR.

**Data: **Text vectorization matrix $X{\rm \; }\epsilon {\rm \; }R_ + ^{{\rm \; \; }m \times n}$, the number of topics (*K*)
1 define $ave\_dis$ list (*L*)
2 **for** each $k\; \epsilon \; \left\{ {2, \ldots ,\; K\; } \right\}$ **do**
3 text classification
4 define diameter list (*L1*)
5 **for** each ${k_i}\; \epsilon \; \left\{ {1, \ldots ,\; k\; } \right\}$ **do**
6 calculate ${D_{{k_i}}}$ by Eq. (3)
7 *L1* append ${D_{{k_i}}}$
8 **end**
9 define diameter list (*L2*)
10 **for** each ${D_{{k_p}}}$ $\epsilon \; \left\{ {L{1_1}, \ldots ,\; L{1_{m - 1}}} \right\}$ **do**
11 **for** each ${D_{{k_q}}}$ $\epsilon \; \; \left\{ {L{1_2}, \ldots ,\; L{1_{m - 2}}} \right\}$ **do**
12 calculate ${D_{p,q}}$ by Eq. (3)
13 calculate $D\left( {p,q} \right)$ by Eq. (5)
14 *L2* append $D\left( {p,q} \right)$
15 **end**
16 **end**
17 calculate $ave\_dis\left( k \right)$ by Eq. (6)
18 *L* append $ave\_dis\left( k \right)$
19 **end**
20 calculate $AICDR\left( k \right)$ by Eq. (7)

## The workflow of aicdr

**Figure 3:**
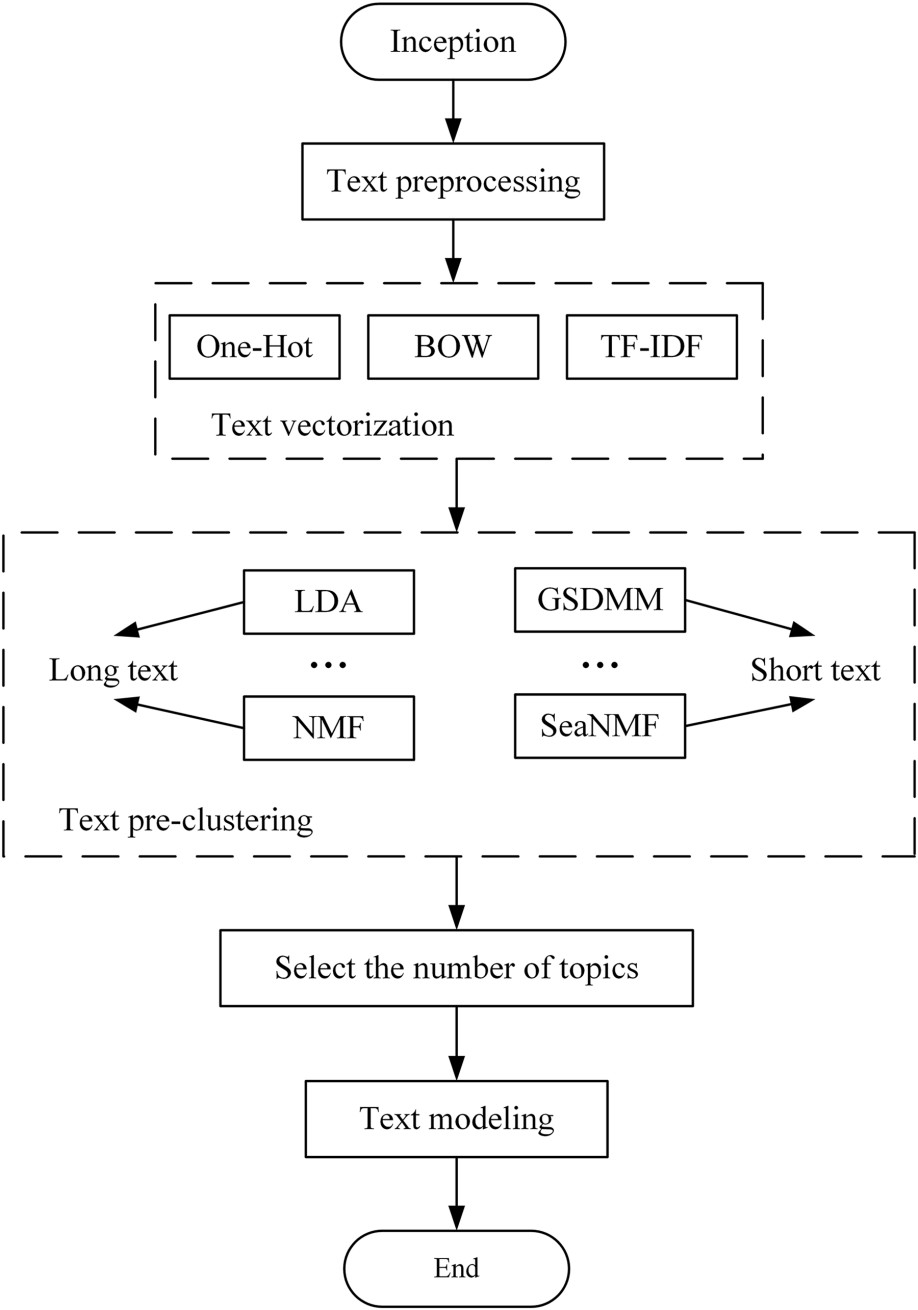
Selection of the number of topics flow chart.

AICDR is a method for selecting the number of topics based on clustering results. Firstly, it is necessary to apply the topic model to a complete dataset and obtain clustering results under different $K$ values, $K\epsilon {\rm \; }\left[ {{K_{min}},{K_{max}}} \right]$. Then, use AICDR to determine the optimal number of topics and re-model. The overall process of selecting an appropriate number of topics based on AICDR is presented, which includes text preprocessing, text vectorization, text pre-clustering, selecting the number of topics, and topic modeling. Figure 3 shows the complete process of selecting the number of topics.

### Text preprocessing

**Figure 4:**
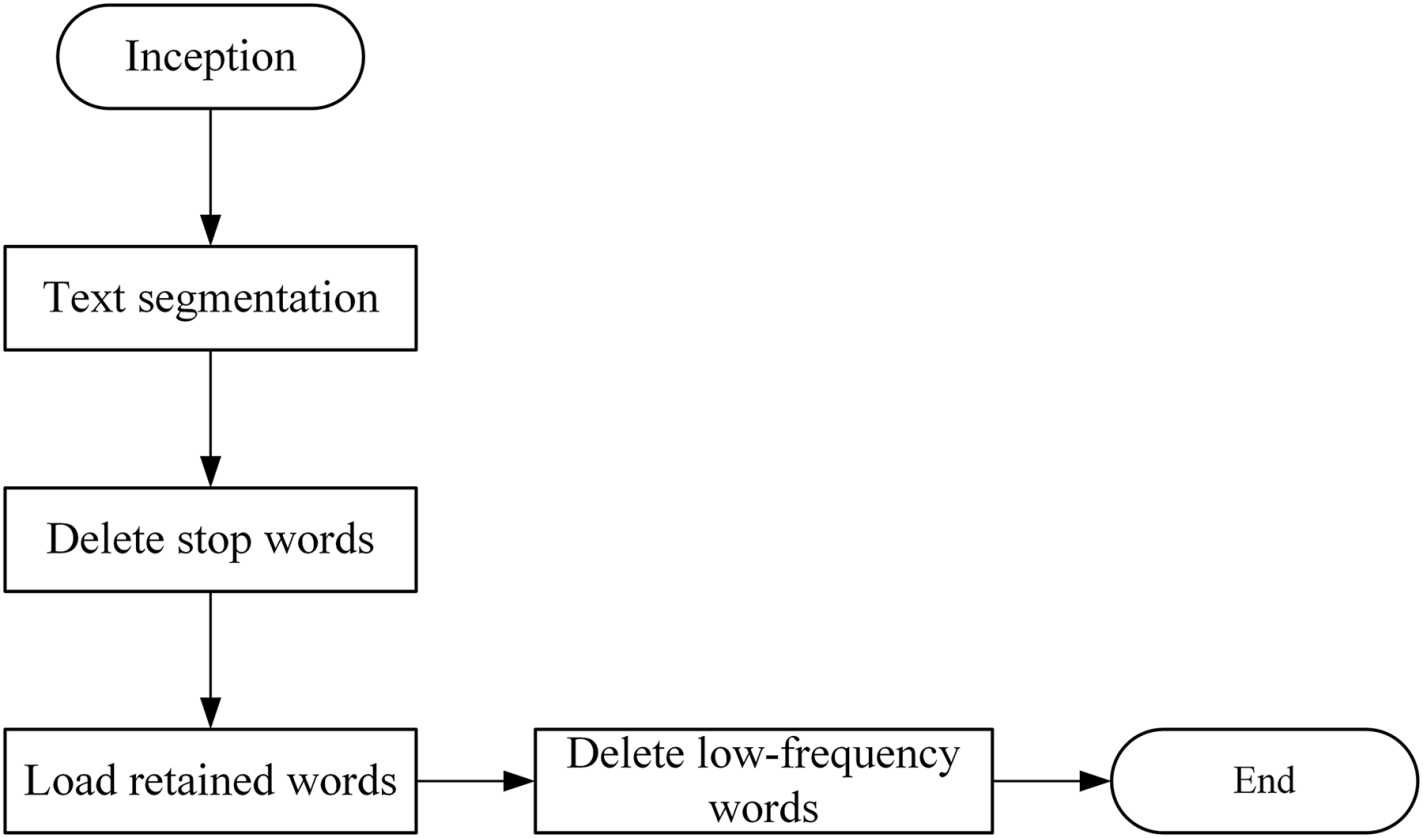
Text preprocessing steps.

Due to the large amount of data, noise, and other characteristics of text datasets, text preprocessing is required before topic modeling. It can filter out invalid information to improve the extraction accuracy of the core keywords. As shown in Fig. 4.

Text segmentation: In English, words are separated from each other by spaces, so English word separation is relatively simple. In Chinese, the text is usually separated using “Jieba” participles. “Jieba” separates the text precisely without redundant words, which better summarizes and expresses the topic and content of the text.

Delete stop words: There are lots of words in the text that have no practical meaning. These words are called stop words, such as “we”, “of”, “yes” and so on. Excessive stop words diminish the model’s ability to generalize and increase computational costs. Therefore, some meaningless stop words should be deleted.

Load retained words: Contrary to stop words, retained words highlight key features of the text. These words should not be separated in the text. Such as “give up”, “hold on” and “all in all”. These words are usually phrases, and once separated, they lose their original meaning.

Delete low-frequency words: Based on data quality considerations, low-frequency words have a small amount of text and have a relatively small impact on classification results. Removing low-frequency words reduces the sparsity of data and reduces the consumption of computing resources. Therefore, delete words with a frequency below a certain threshold.

### Text vectorization

Salton, Wong & Yang (1975) proposed vector space model (VSM), which converts each text into a certain vector, and the text dataset is transformed into a high-dimensional vector space. In the process of text vectorization, the text is broken down into smaller units, which can be words, phrases, and other semantic units. For a text dataset, we need to construct a vocabulary, where each word in the vocabulary has a unique index. Each text is represented with $t = \left( {{w_1},{w_2},{w_3}, \ldots ,{w_m}} \right)$. Here t means a text, ${w_i}$ means the weight of a word. The text dataset is viewed as a matrix, $D = \left( {{t_1},{t_2},{t_3}, \ldots ,{t_n}} \right)$.

#### One-Hot

One-Hot weighs a value of 0 or 1 to each word. If there is a word in the text, its weight is 1, otherwise it is 0. Despite the simplicity of the one-hot method, it is hard to capture the importance of words.

#### BOW

Bag-of-words (BOW) would similarly represent each text as a vector, where each element of the vector corresponds to a word in the vocabulary, and its value is the number of times the word occurs in the text. This method considers word frequency information but ignores word importance and fails to identify false keywords.

#### TF-IDF

TF-IDF approach evaluates the significance of terms in a document by integrating two metrics: term frequency (TF), which reflects how often a term appears in a document, and inverse document frequency (IDF), which adjusts for the term’s rarity across the *corpus*. Compared with BOW, TF-IDF has a strong ability to recognize false high-frequency words and redundant words, it is robust to sparse and unstructured data. Therefore, it is more advantageous in word weight assignment and noise suppression.

### Text pre-clustering and selection of topic numbers

AICDR is calculated based on clustering results, and each text in the dataset needs to have a stable cluster label.

We have introduced four different topic models. LDA and NMF are suitable for short texts, while GSDMM and SeaNMF are good for long texts. The four models will be applied to multiple corpora for text clustering, with the resulting classifications utilized for subsequent AICDR calculation. When AICDR reaches its maximum value, the corresponding *K* is the optimal number of topics. Topic modeling is performed based on the optimal number of topics again.

## Experiments

In this section, we evaluate the proposed AICDR algorithm with relevant experiments and analyze it in comparison with other methods for selecting the number of topics, respectively. The whole experiment is executed on a CPU of Intel® Core™ i5-8300H.

### Experiment datasets

We assess the effectiveness of our proposed approach using a variety of authentic textual data collections, which are detailed subsequently:

- BBCnews (Greene & Cunningham, 2006): This dataset contains 2,225 documents from the BBC news site, spanning five categories: business, entertainment, politics, sport, and tech, from 2004 to 2005.
- BBCsport (Greene & Cunningham, 2006): The dataset includes 737 sports articles from BBC, covering athletics, cricket, football, rugby, and tennis, from 2004 to 2005.
- Reuters (Joachims, 1998): The Reuters dataset, sourced from 1,987 financial news. We have selected 1,899 documents across three categories for our analysis.
- AGNews (Lecun, 2015): We have filtered 7,409 articles from AG news, categorized as: world, sport, business, and tech.
- Snippets: The dataset is from web search results, divided into Snippets-1 with 7,870 queries in business, computers, arts, and education, and Snippets-2 with 4,496 queries in engineering, health, politics, and sports.

The basic description of the dataset is shown in Table 2.

**Table 2:** Description of experimental datasets.

Datasets	Clusters	Doc	Word	Balance (%)
BBCnews	5	2,225	8,835	75.54
BBCsport	5	737	3,272	37.74
Reuters	3	1,399	3,440	91.50
AGNews	4	7,409	8,063	96.53
Snippets-1	4	7,870	6,294	56.39
Snippets-2	4	4,469	4,120	24.60

### Comparison methods

Here are several topic models and three methods of selecting the number of topics introduced.

#### Methods of selecting the number of topics

- Elbow method. The elbow method is commonly used in cluster analysis to determine the optimal number of clusters, particularly suitable for the K-Means clustering algorithm.
- AQDEB (Shi et al., 2021). When the SSE curve is quite smooth, it is difficult to identify the inflection point. A quantitative discriminant method of elbow point (referred to as AQDEB) effectively has solved this problem. The index of the minimal inter-section angles between elbow points is used as the estimated potential optimal cluster number.
- Stability analysis (Greene, O’Callaghan & Cunningham, 2014). The term-centric stability analysis strategy can efficiently determine the appropriate number of topics while being more applicable to a wider range of topic models and classification methods. It evaluates the consistency between the ranking term lists of topic models generated under different data samples.

We have provided parameter explanations for some methods. To apply these methods, we first need to use various topic models to generate clustering results of text under different K values. Compared to the other two algorithms, elbow method is relatively simple. It evaluates the within-cluster sum of squares (WCSS) of intra-cluster errors under different numbers of clusters, and plots the relationship between $K$ value and WCSS to find the position of the “elbow” in the curve, without the need for additional parameter settings. For AQDEB, it is an improvement based on the elbow method that does not require additional parameters. For stability analysis, the depth is 10, we only focus on the top 10 ranked words. Then ${\rm \; }\tau = 10$, extract only 10 times from each completed dataset. The ratio of the sample dataset to the complete dataset is 0.8. Due to the long computation time of stability analysis, the range of the number of topics is set to 2 to 10, $K \; \epsilon {\rm \; }\left[ {2,10} \right]$. The range of topic numbers for other methods is 2 to 20, $K \; \epsilon {\rm \; }\left[ {2,20} \right]$.

Although the methods based on the elbow method are more suitable for K-Means, they are all based on clustering results for selecting the number of topics. Therefore, we applied the three comparison methods and AICDR to different topic models and K-Means algorithms.

#### Brief description of topic models

In the previous section, we have introduced four topic models and three text vectorization methods. Therefore, we’ll further provide simplified usage descriptions for various models.

LDA and NMF are suitable for short texts, while GSDMM and SeaNMF are good for long texts. Meanwhile, different topic models may use different vectorization methods. LDA and GSDMM adopt BOW, while NMF and SeaNMF adopt TF-IDF. The four models will be applied to multiple corpora for text clustering, with the resulting classifications utilized for subsequent AICDR calculation. Due to the elbow bending method being used as a comparison method, we will also apply the more suitable K-Means for long text classification. K-Means also uses TF-IDF.

### Clustering accuracy

The performance of different models may vary on different datasets, such as limitations on text length. To select a more suitable number of topics, we need topic models with better performance. We only consider the clustering accuracy of the algorithm here. The evaluation indicators of topic models usually include topic coherence, topic stability, and topic interpretability (Lau, Newman & Baldwin, 2014). AICDR selects the number of topics based on the classification results of the dataset. Therefore, we only need to consider applying the topic model to document classification, and evaluate the clustering accuracy of the model using standardized mutual information (NMI), automatic readability index (ARI), and accuracy (ACC). Table 3 shows the performance of different models on different datasets.

**Table 3:** The clustering accuracy of different models on the correct number of topics. The best results are highlighted in bold (The higher the better).

Datasets	Topic models	Metrics		
		NMI	ARI	ACC
BBCsport	LDA	0.709	0.662	0.851
	NMF	0.818	0.856	0.872
	K-Means	**0.894**	**0.896**	**0.963**
BBCNews	LDA	0.727	0.701	0.862
	NMF	**0.812**	**0.842**	**0.932**
	K-Means	0.751	0.726	0.868
Reuters	LDA	0.432	0.443	0.669
	NMF	0.550	0.597	0.776
	K-Means	**0**.**632**	**0.671**	**0.832**
AGNews	GSDMM	**0.585**	**0.622**	**0.833**
	SeaNMF	0.563	0.600	0.822
Snippets-1	GSDMM	0.565	0.592	0.830
	SeaNMF	**0.580**	**0.634**	**0.850**
Snippets-2	GSDMM	0.764	0.823	0.919
	SeaNMF	**0.787**	**0.850**	**0.939**

Two methods are used to select the number of topics based on SSE, which can also be applied to centroid-based clustering algorithms, such as K-Means. Thus K-Means is introduced for text classification. Overall, K-Means performs well on long text datasets, especially on BBCsport and Reuters. NMF ranks second, with good performance on BBCNews. LDA performs relatively poorly. In short text datasets, SeaNMF has relatively high clustering accuracy, but the difference between GSDMM and it is not significant. Therefore, both can effectively perform short text clustering. Due to the sparsity inherent in short texts, the performance of K-Means is poor; hence, the clustering of short texts by K-Means is not presented here.

### Results analysis

To assess the precision in determining the number of topics and the adaptability to diverse topic models, we compared AICDR with three other methods for establishing the optimal K across various datasets and topic models, as detailed in Table 4.

**Table 4:** The optimal number of topics is determined by different methods for different topic models. The best results are highlighted in bold.

Datasets	Topic models	Methods			
		Elbow method	AQDEB	Stability analysis	AICDR
BBCsport	LDA	13	13	**5**	**5**
	NMF	3	6	4	4
	K-Means	**5**	13	**5**	**5**
BBCNews	LDA	3	17	**5**	6
	NMF	3	19	2	**5**
	K-Means	3	16	6	6
Reuters	LDA	**3**	12	**3**	**3**
	NMF	**3**	7	4	**3**
	K-Means	**3**	13	**3**	**3**
AGNews	GSDMM	3	19	3	**4**
	SeaNMF	3	16	3	5
Snippets-1	GSDMM	**4**	12	**4**	**4**
	SeaNMF	18	**4**	**4**	**4**
Snippets-2	GSDMM	3	19	3	3
	SeaNMF	3	11	3	3

The experimental results highlight the effectiveness of determining the number of topics based on the AICDR method. Even on Snippets-1 and Reuters, AICDR based on different topic models can determine the optimal number of topics. For BBCsport, BBCNews, and AG-News, AICDR can effectively determine the number of topics in some topic models. Although it does not determine the optimal number of topics on other models, the difference from the optimal number of topics is not significant. Although the number of topics it determines on other models is not optimal, the difference from the optimal number of topics is generally minimal, the difference is usually 1. Referring to Table 3, BBCsport, BBCNews, and AG News showed better clustering accuracy when applied with K-Means, NMF, and GSDMM, respectively. Thus, the better the performance of the topic model (clustering accuracy), the higher the precision of the AICDR.

Elbow method only shows accurate performance on Reuters, with relatively poor performance on other datasets. However, the number of topics it determines is also close to the number of clusters in other datasets. Meanwhile, it primarily judges the elbow point of the SSE curve, this method can accurately determine the number of topics when applied with K-Means on both BBCsport and Reuters datasets. Elbow method is indeed more suitable for selecting $K$ in K-Means.

AQDEB does not exhibit particularly outstanding performance across all datasets. It employs the arccosine theorem to compute the inter section angles between elbow points, the index of minimal inter section angles between elbow points is used as the estimated potential optimal cluster number. The mean distortion curves indicate that the index does not decrease completely with the increase of K, *i.e*., the curves are not fairly smooth at lower $K{\rm \; }$ values. This also explains why the number of topics determined by AQDEB is more concentrated at the lower $K$ values.

The number of topics determined based on Stability analysis is relatively accurate. This method does indeed show a certain level of adaptability and can effectively be used to determine the optimal number of topics for some models. When the determined number of topics is not optimal, it can still approximate the original number of clusters in the dataset, which can also be maintained at the same level as AICDR. But overall, its accuracy is slightly lower than AICDR.

**Figure 5:**
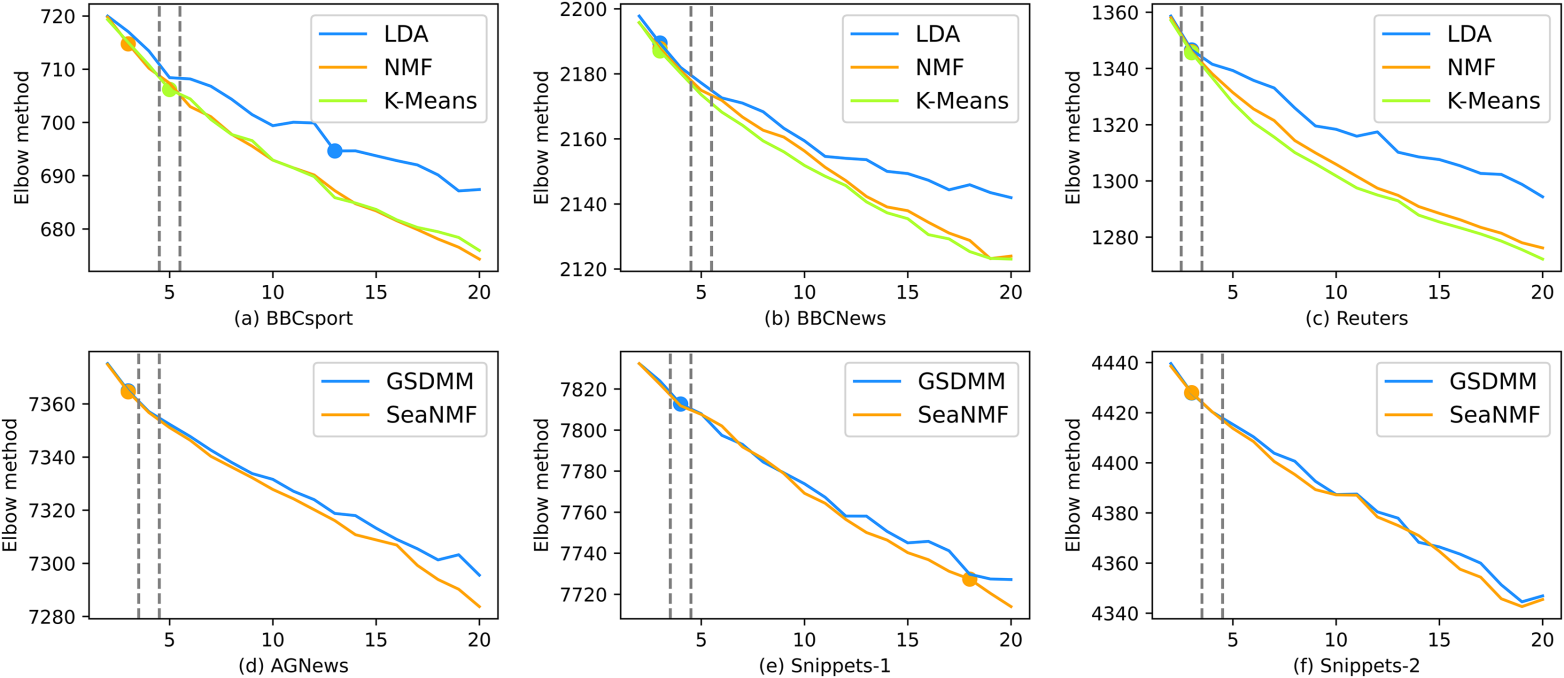
The performance of the elbow method.

**Figure 6:**
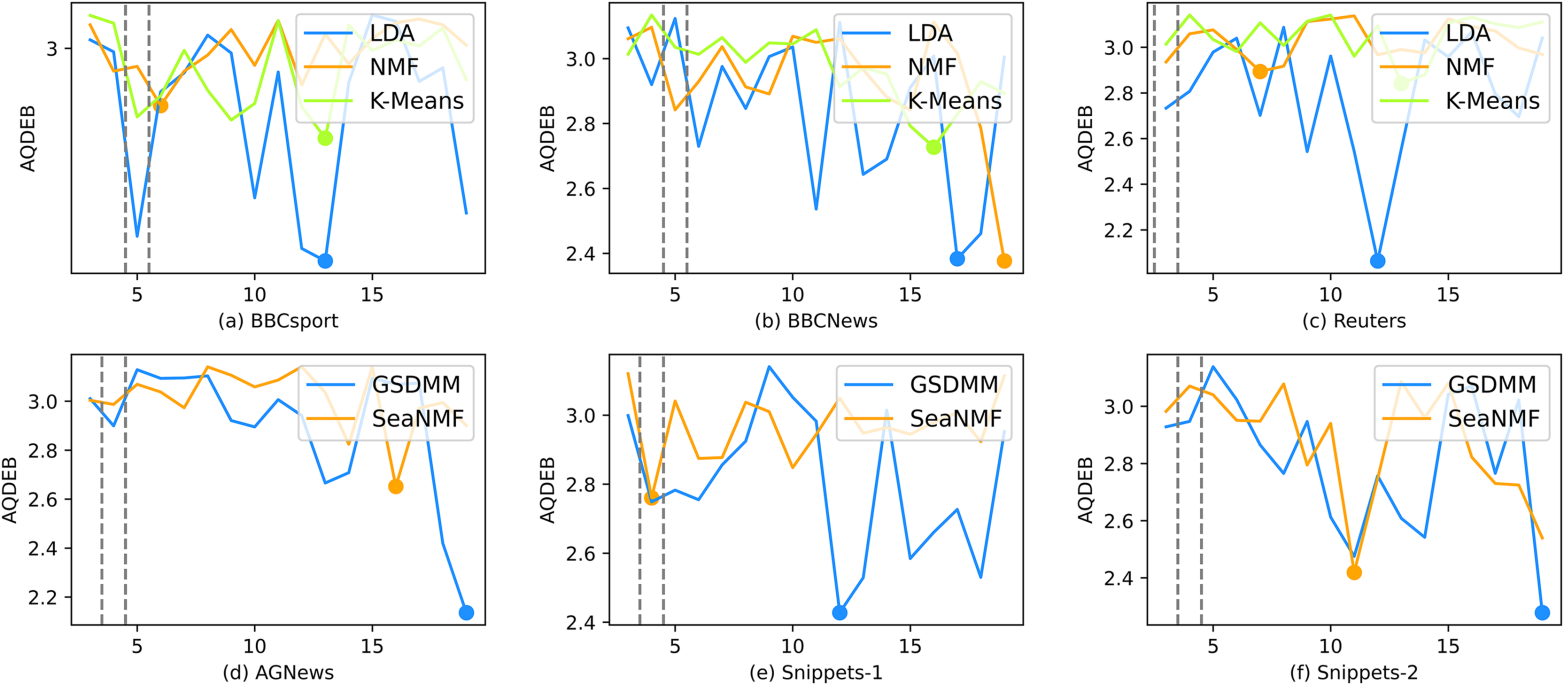
The performance of the AQDEB.

**Figure 7:**
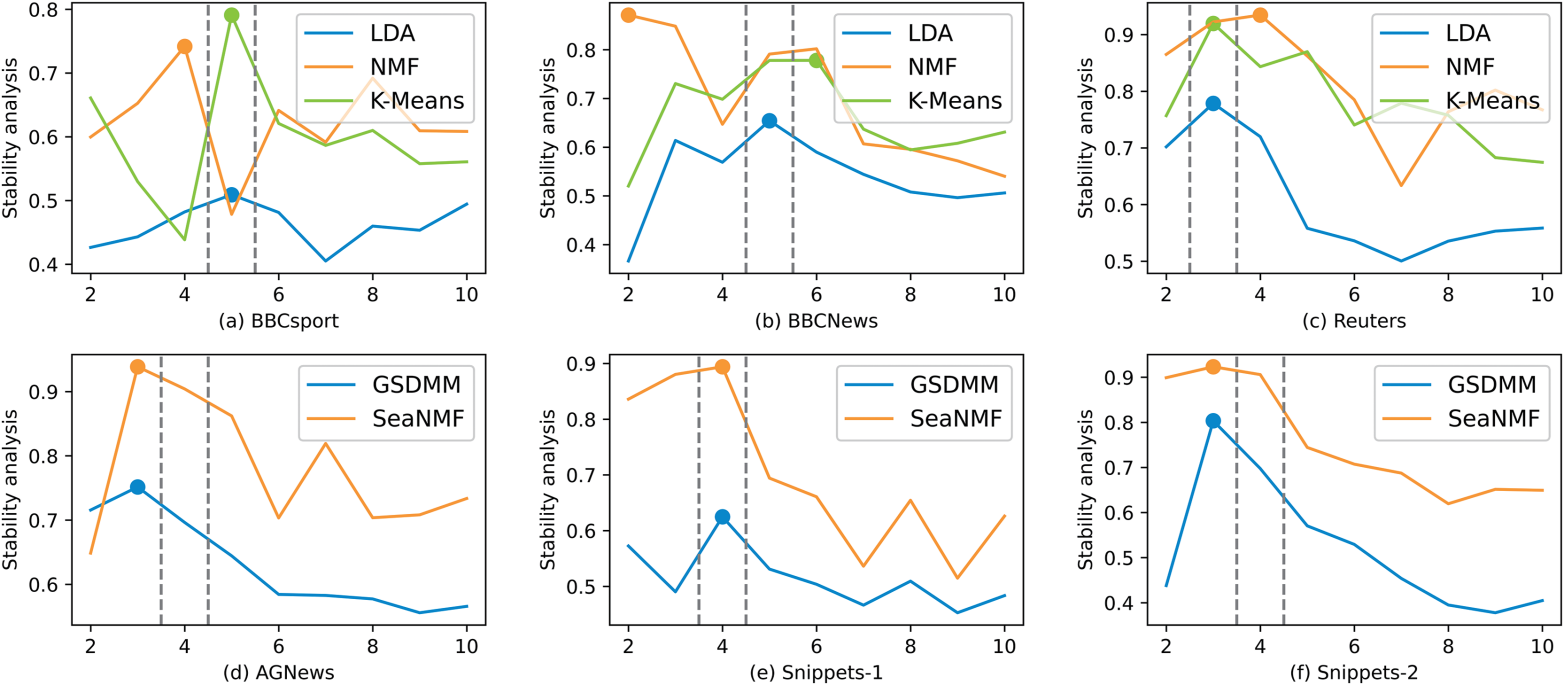
The performance of the stability analysis.

**Figure 8:**
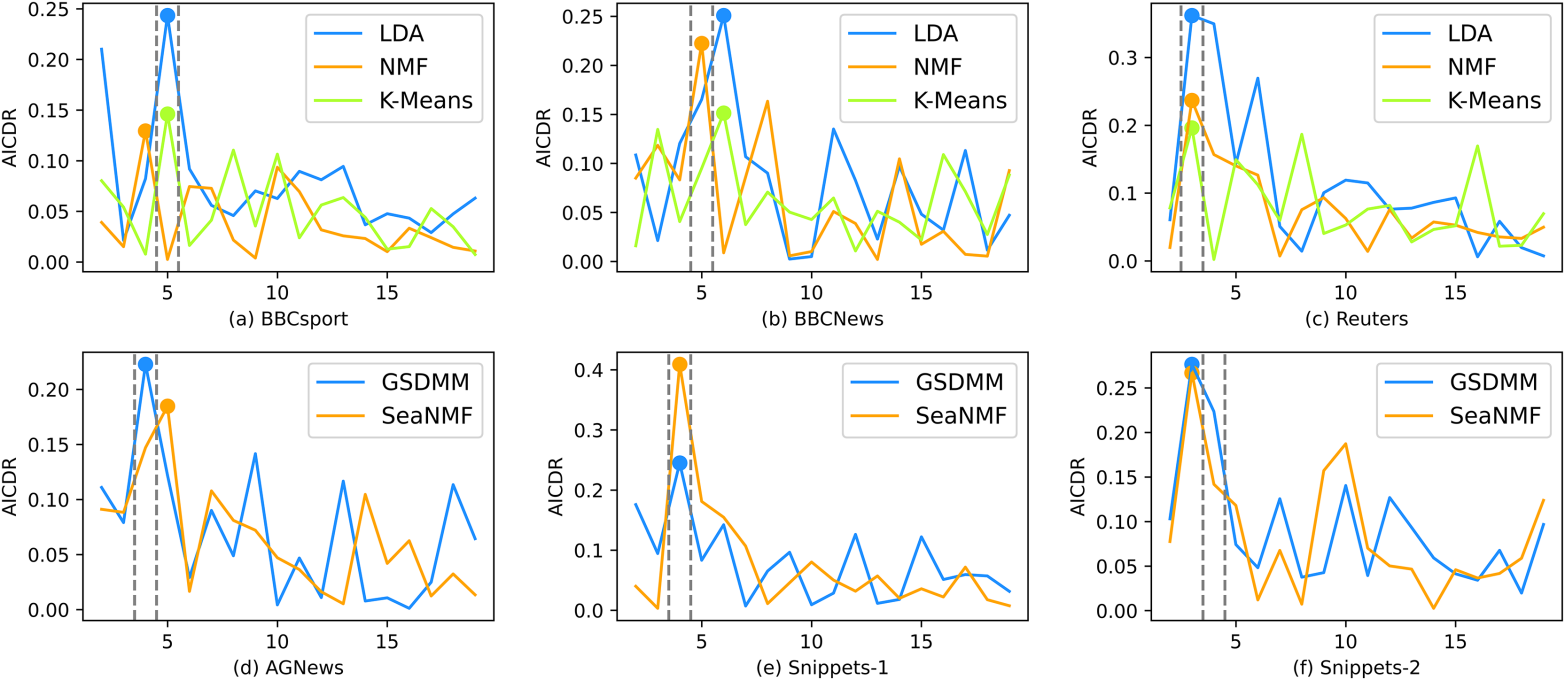
The performance of the AICDR.

As shown from Figs. 5 to 8, the variation of different indicators with $K$ is presented. As the number of topics increases, AICDR exhibits a fluctuating decrease, akin to undulating peaks and troughs, this indicates that the inter-class distance also decreases as $K$ increases The corresponding AICDR for The neighbor of the optimal $K$ is also relatively high. For elbow method, the variation of SSE with $K$ is presented and the elbow point (the optimal number of topics) is marked. AQDEB is an improved method for determining the elbow point. It is obvious that the SSE curve does not decrease completely smoothly at lower $K$ values, so the optimal number of themes judged by AQDEB is mostly concentrated at lower $K$ values. Stability analysis is similar to AICDR in that its values are relatively high around the correct number of topics. This further demonstrates that stability analysis and AICDR indeed have excellent ability to identify the optimal number of topics, as well as strong adaptability to various models.

Overall, AICDR can identify the optimal number of topics across all datasets by applying different topic models or clustering algorithms. Mainly, it is not limited by topic models or clustering algorithms and has good adaptability to most methods. Notably, the higher the clustering accuracy of the model, the stronger the identification capability of AICDR.

### Stability analysis

Stability analysis is most commonly conducted by perturbing the data, which involves generating sub-samples through random sampling of the original objects. By doing so, we can assess the consistency and reliability of AICDR across different subsets of the data, ensuring its robustness and effectiveness in various scenarios. Here, subsets of the dataset are taken at 70%, 80%, and 90% respectively. During the sampling process, no random seeds are set to ensure the randomness of the subset.

**Figure 9:**
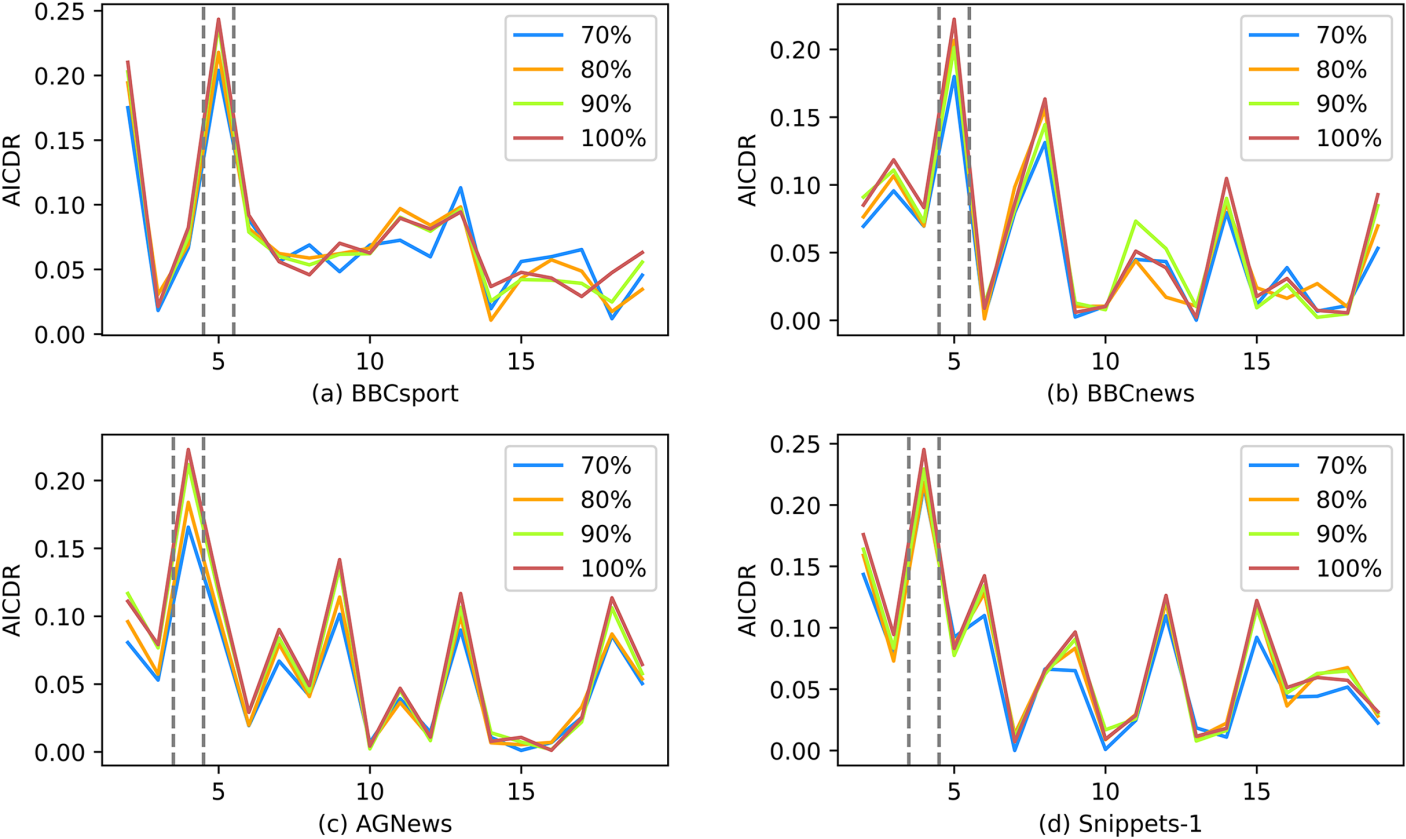
The performance of AICDR on different subsets.

Figure 9 shows the performance of AICDR on subsets of four datasets. From the figure, it can be observed that the AICDR curve exhibits high consistency across different subsets and the entire dataset. Although there are slight fluctuations within certain *K* value ranges, overall, the number of topics determined by AICDR on different subsets is completely accurate. This indicates that AICDR has excellent robustness and stability.

### Preprocessing and vectorization in AICDR

In this section, we use different text vectorization methods and set different low-frequency words to explore the influencing factors of AICDR. The effects of low-frequency words and text vectorization on AICDR are shown in Tables 5 and 6.

**Table 5:** The impact of low-frequency words on the performance of AICDR. The best results are highlighted in bold.

Datasets	Topic models	Methods		
		One-Hot	BOW	TF-IDF
BBCsport	LDA	**5**	**5**	**5**
	NMF	4	4	4
	K-Means	**5**	2	**5**
BBCNews	LDA	6	6	6
	NMF	**5**	**5**	**5**
	K-Means	3	3	6
Reuters	LDA	**3**	**3**	**3**
	NMF	**3**	**3**	**3**
	K-Means	**3**	2	**3**
AGNews	GSDMM	**4**	**4**	**4**
	SeaNMF	5	5	5
Snippets-1	GSDMM	**4**	**4**	**4**
	SeaNMF	**4**	**4**	**4**
Snippets-2	GSDMM	**3**	**3**	**3**
	SeaNMF	**3**	**3**	**3**

**Table 6:** The impact of text vectorization on the performance of AICDR. The best results are highlighted in bold.

Datasets	Topic models	Number of words		
		5	10	15
BBCsport	LDA	**5**	**5**	**5**
	NMF	4	4	4
	K-Means	**5**	**5**	**5**
BBCNews	LDA	6	6	6
	NMF	**5**	**5**	**5**
	K-Means	6	6	6
Reuters	LDA	**3**	**3**	**3**
	NMF	**3**	**3**	**3**
	K-Means	**3**	**3**	**3**
AGNews	GSDMM	**4**	**4**	**4**
	SeaNMF	5	5	5
Snippets-1	GSDMM	**4**	**4**	**4**
	SeaNMF	**4**	**4**	**4**
Snippets-2	GSDMM	**3**	**3**	**3**
	SeaNMF	**3**	**3**	**3**

In Table 5, different vectorization methods have a weak impact on AICDR, while One-Hot, BOW, and TF-IDF have no significant effect on AICDR overall. However, when the classification model is K-Means, One-Hot, and BOW reduces the performance of AICDR, and the number of determined topics deviates greatly from the original number of topics in the *corpus*. This influence appears in the long text *corpus*. When the vectorization method is TF-IDF, AICDR exhibits the best performance regardless of the topic model. Therefore, TF-IDF and AICDR are more compatible. In Table 6, low-frequency words have little effect on AICDR. We set the thresholds for low-frequency words to 5, 10, and 15, while the number of topics determined by AICDR remained consistent. Therefore, low-frequency words have no significant impact on AICDR.

Overall, while preprocessing techniques and vectorization methods generally have limited impact on AICDR, the TF-IDF vectorization method demonstrates greater compatibility with AICDR.

## Conclusion

A key challenge when applying topic modeling is the selection of an appropriate number of topics $K$. In this article, we propose a selection method for determining the number of topics based on inter-class distance, named average inter-class distance change rate (AICDR). By calculating the AICDR for consecutive $K$ values, the optimal number of topics is selected as the previous $K$ value when the difference between the two is maximized. The optimal clustering result, with a specified number of topics, should exhibit high intra-class similarity and low inter-class similarity. AICDR considers the inter-class distance comprehensively, which is computed by the diameter of the class, and avoids topic overlap, improves intra-class similarity, and reduces inter-class similarity. Meanwhile, it is not limited by topic models or clustering algorithms and can effectively determine the number of topics in most methods. Evaluations on several real text datasets have suggested that AICDR can provide a useful guide for selecting the optimal number of topics.

In upcoming research endeavors, we mainly focus on improving the robustness and stability of AICDR to noise. Meanwhile, we will explore other potential limitations of AICDR and alleviate these limitations to improve the performance of AICDR.

## Supplemental Information

10.7717/peerj-cs.2723/supp-1 Supplemental Information 1 Topic models, AICDR, and other comparative methods.

10.7717/peerj-cs.2723/supp-2 Supplemental Information 2 Dataset.

## Additional Information and Declarations

### Data Availability

The following information was supplied regarding data availability:

The code is available at GitHub and Zenodo:

- https://github.com/cjluxy/AICDR-Selecting-the-number-of-topics.

- cjluxy. (2025). cjluxy/AICDR-Selecting-the-number-of-topics: Python code and data for AICDR (AICDR-v1). Zenodo. https://doi.org/10.5281/zenodo.14784034.

The BBCnews dataset and BBCsport dataset are available at http://mlg.ucd.ie/datasets/bbc.html.

The Reuters dataset is available at https://martin-thoma.com/nlp-reuters.

The AGNews dataset is available at Kaggle: https://www.kaggle.com/datasets/amananandrai/ag-news-classification-dataset.

The Snippets1 dataset is available at Zenodo: Xu, Y. (2025). Snippets1 [Data set]. Zenodo. https://doi.org/10.5281/zenodo.14822881.

The Snippets2 dataset is available at Zenodo: Xu, Y. (2025). Snippets2 [Data set]. Zenodo. https://doi.org/10.5281/zenodo.14822895.

The data is available in the Supplemental File.
